# Tonics*Dr. Waugh writes: “I am enclosing herewith a short paper upon ‘Tonics,’ especially designed to call the attention of the profession to the danger which they run in prescribing alcoholic preparations. I have had one such case, which made such an impression upon me that I feel it advisable to call professional attention to this matter.”

**Published:** 1911-09

**Authors:** William F. Waugh

**Affiliations:** Professor of Therapeutics, Bennett Medical College, Chicago, Ill.


For Texas Medical Journal.

### Tonics.*

BY WILLIAM F. WAUGH, A. M., M. D.,
Professor of Therapeutics, Bennett Medical College, Chicago, Ill.

He feels weak, languid, run-down, has no appetite; work is a burden, and pleasure has lost its charm. So he goes to the drug store and asks for a tonic. The obliging pharmacist knows exactly what is needed, and proceeds to concoct a mixture of various ingredients, such as may be represented by the elixir of quinine, strychnine, iron, etc. The man takes a dose before each meal, and finds it good to the taste and tonic to the stomach; it makes him hungry, and there is a sense of vigor after each dose. Quite the thing! Well worth 50 cents—and saved a doctor's fee.

Query—Which would confer the greatest boon upon humanity, obliteration of the tubercle bacillus, or extinction of the custom of charging a patient every time he consults the doctor? Let the man pay his medical adviser by the year, and consult him whenever he chooses.

Suppose he does come to the doctor with the train of symptoms above sketched. The physician wants to know something more—why the symptoms? The man has been eating too much, or improper food, and his stomach has wisely called a halt. His bowels have become clogged, and a fecal strain in his blood is poisoning the digestive organs, and the nerve centers are being hurt. The kidneys are out of whack, and their vital depuration is imperfect. Overwork, overworry, underrest, underexercise, bad hygienic influences about the house, sewer gas in the bedroom, too little fresh air, a dead rat behind the plastering; these are a few of the things for which our man takes "elixir, quinine, strychnine, ferri," etc.

What folly! Were this the typic application of drugs we would cheerfully subscribe to the nihilistic creed, and cry, away with them!

There's another, and more serious matter, one that every last

---

*Dr. Waugh writes: "I am enclosing herewith a short paper upon 'Tonics,' especially designed to call the attention of the profession to the danger which they run in prescribing alcoholic preparations. I have had one such case, which made such an impression upon me that I feel it advisable to call professional attention to this matter."

one of us knows, but to which scarcely any give sufficient attention. Note the sense of exhilaration following the dose of tonic, the patient's conviction that the remedy has "touched the spot." That's not iron, quinine or nux—it's alcohol. He feels the good effect and he wants more. Needs it at lunch time, but the bottle is at home, so he takes a cocktail and gets the same exhilaration, the same access of appetite and digestive strength.

It is a just reproach to us that some inebriates are started in the alcoholic way by our prescriptions; and this is the worse for us because we do not need to do it. Setting aside the moot question of whether alcohol is ever essential and unavoidable in medical practice—we take the negative—in the majority of cases where it is prescribed it is negligently, without a thought of the danger of the habit. If you want to prescribe whisky, do so; say "whisky" openly and aboveboard, that people may know what they are doing. But don't disguise it under medical terms, and let men form the alcohol habit innocently, unwittingly.

One such case is a warning—one that worries the doctor's conscience for many a year. But why that one case? We know very well that there are some persons in whom the slightest taste of alcohol suffices to awaken the sleeping devil in them. We know that many more can form the habit readily, since the stimulation of one dose is followed by a reactive depression that calls for another.

In prescribing, absolutely divorce alcohol from other drugs. Give alcohol if you must, for distinct indications, but never as an excipient. Use water-soluble agents whenever possible—and there are very few instances in which any other fluid menstruum is as desirable. The pure alkaloid diffused through a measured quantity of water in a glass, just enough to last till the next visit, is the ideal in the usual run of cases. Clean-cut, careful prescribing, comes from and leads to clean-cut, careful diagnosis. Mix up the alcoholic element with the remedy you want, and exactness is lost, and possibilities of evil begin.

